# Highland Malaria Transmission Dynamics in Space and Time Before Pre-elimination Era, Northwest Ethiopia

**DOI:** 10.1007/s44197-022-00034-8

**Published:** 2022-03-27

**Authors:** Yalemwork Ewnetu, Wossenseged Lemma

**Affiliations:** 1 Department of Medical Biotechnology, Institute of Biotechnology, University of Gondar, Gondar, Ethiopia; 2 Gondar University Specialized Hospital, University of Gondar, Gondar, Ethiopia; 3 Department of Medical Parasitology, College of Medicine and Health Sciences School of Biomedical and Laboratory Sciences, University of Gondar, Gondar, Ethiopia

**Keywords:** Highland malaria, Malaria transmission dynamics, Malaria elimination program, Northwest Ethiopia

## Abstract

**Background:**

Currently, the district-level malaria transmission stratification has indicated the Northern, Northwestern, Southern, and rift valley lowland and surrounding highland districts are almost entirely classified as high or moderate malaria transmission zones. Conducting malaria surveillance to track, test, and treat all malaria cases cannot be implemented in Ethiopia in the current situation.

**Objective:**

To show malaria transmission dynamics in different health facilities located from 1800 to 2772 m altitudes during 2018–2021 in Northwest Ethiopia.

**Methods:**

A total of 3.5 years (2018–2021) retrospective confirmed and treated malaria cases in 43 kebeles health posts and clinics in Gondar Zuria district were used for analysis.

**Result:**

The total malaria count was 5893 for 2019 compared to 31, 550 for 2020 and 33, 248 for 2021. Mean monthly malaria incidence/1000 people in 2019 was 2.39 ± 5.4 and increased to 10.64 ± 16.99 in 2020 and 11.19 ± 16.59 in 2021. Annual malaria incidence increased from 24 cases/1000 people in 2019 to 139.08 cases/1000 people in 2021 and is alarming danger in malaria elimination program in the district or the country as a whole. Poisson and Negative binomial regressions models indicated 5.78- and 5.26-fold malaria cases increase, respectively, in 2021 compared to 2019. The sudden increase in malaria incidences (counts) in 2020 and 2021 coincided with the interruption of residual insecticide application in Gondar Zuria district during the transition period towards the malaria pre-elimination stage implicating the role of malaria control tools in suppressing transmission. Study on climate variability also indicated that the rainfall variability in different months might have also favored high malaria transmission in 2020 and 2021 compared to 2019. Thus, in addition to re-starting the use of malaria control tools, giving attention to climate anomalies (variability) that favors malaria transmission, for prompt interventional actions, is required. The malaria elimination program in Ethiopia might have not reached a pre-elimination stage as malaria cases per 1000 people have not decreased below five in the majority of Ethiopian districts. Tracing, confirming, and treating individual cases to stop further transmission is, almost, impossible. In a situation like this, the Ethiopian malaria elimination program should work intensively towards understanding malaria epidemiology at the district level to re-design a localized malaria control strategy. The renewed malaria control program should also consider altitudes above 2000 m.

## Introduction

Following the success history of the Roll Back Malaria initiative in global malaria burden reduction using insecticide-treated nets (ITNs) in 2010, the interest to eliminate malaria from the globe has increased [1]. In 2015, the Global malaria elimination strategy (2015–2030) was endorsed to create a malaria-free world [2]. In an effort of the malaria-free world in 2030, World Health Organization (WHO) has the vision to reduce malaria mortality and incidence at least by 40% in 2020, and 75% by 2025, and 90% by 2030 including step by step malaria elimination from at least 10 countries in 2020 and 20 countries in 2025 before the final 35 countries in 2030 [2]. Malaria elimination is the interruption of local transmission by reduction to zero incidences while eradication is a permanent reduction to zero [3]. Despite of all the efforts made to eliminate malaria, the number of malaria cases reported globally remained high. Globally, there were an estimated 229 million malaria cases in 2019 in malaria-endemic countries compared to 228 million cases in 2018 with Africa sharing 93% and 94% of these cases, respectively [1, 3]. Recent report has indicated, further, increase of malaria cases in 2020 (241 million) compared to 2019 (WHO, 2021). About 51% of all global cases were reported from Nigeria (27%), the Democratic Republic of the Congo (12%), Uganda (5%), Mozambique (4%), and Niger (3%) in the year 2019 [1]. Although Ethiopia was among African countries, which showed a 40% reduction and a promise to achieve the 2030 malaria elimination goal [1], the recent two-fold increase in malaria cases (1, 743, 755) in 2020 [4] from what was in 2019 (904, 495) [1] caused a doubt for Ethiopia to remain on the malaria elimination track. Unless African countries which are completely and temporarily off the track of malaria transmission work hard in addition to those on the track of elimination, malaria will not be a history from Africa or the world. Malaria transmission must be reduced significantly in African countries to reach the pre-elimination stages before it could be possible to detect individual infection (symptomatic/asymptomatic) and immediate treatment to prevent secondary infections in addition to conducting continuous surveillance to prevent the re-establishment of transmission in areas in which it has already been interrupted [5]. Currently, WHO has indicated several challenges during the application of the malaria elimination program which includes inadequate political commitment and leadership, weak malaria program management, insufficient prioritization and sustainability of interventions, inappropriate application of larviciding, inadequate domestic financing, and weak surveillance systems [1]. In northwest Ethiopia, the challenges also include the impact of climate anomalies and the re-introduction of malaria cases to highland areas, from highly malaria-endemic Metema–Humera Northwestern lowlands, due to seasonal migration of adult male laborers [6–9].

Ethiopia is located within 3.30–15°N and 33–48°E in the tropical horn Africa with an area of 1.1 million square kilometers. The different topographic areas are located between the tallest altitude (4, 550 m) at Ras Dashen Mountain to lowest Afar Depression (110 m below sea level). Great East African Rift Valley divides the highlands of Ethiopia into the Northwestern and the Southeastern highlands. Thus, Ethiopia has diverse malaria ecologies in different regions which result in significant variations in malaria prevalence and incidence. Approximately 60% of Ethiopia’s population lives in malarias areas, and 68% of the country’s landmass is favorable for malaria transmission [10]. Complex malaria epidemiology of Ethiopia is related to climatic variability, altitude, topography, presence of water bodies, livelihood style, educational status, gender, economic status, population movement and other interacting Socio-environmental risk factors [9, 11]. Malaria transmission is primarily associated with altitude and rainfall [9, 10]. Gambella and Benshangul Gumuz administration regions, which are almost entirely located in western lowland areas (< 1000 m asl), had the highest malaria prevalence. Parts of Amhara, Oromia and South nation nationality regional states in the western lowland areas also have similar high malaria transmission. Very low malaria transmission is found in the central colder highlands and the arid Somali lowland areas [11].

Ethiopian highlands were characterized by cyclic malaria epidemics until 2003/4 [12–14]. Although Ethiopia has experienced different cyclic catastrophic malaria epidemics due to El-Nino/La-Nina events in highland areas, highland malaria transmission dynamics has not been understood fully. Previous description of malaria transmission dynamics was holistic and was not local malaria epidemiology specific. There was a trend of regarding all malaria transmission as seasonal in addition to considering above 2000 m highland areas as malaria free [10, 11]. As a result, malaria cases found above 2000 m altitude was considered to be imported cases from surrounding endemic lowlands. Generally, highland malaria was defined as unstable malaria transmission in 1500–2000 m asl altitude range (fringe zones) during El-Nino/La-Nino years and associated to anomalous heavy rainfalls and warm temperatures [15–17]. Highland malaria transmission above 2000 m in Ethiopia (East Africa) may not be a result of territorial expansion of malaria due to global warming, as it was described previously [18]. The first reason could be highland malaria epidemics existed in Ethiopia in areas above 2600 m, at least, for the last 60 years [13]. The second reason could be the absence significant mean temperature and rainfall increase in East Africa in the past century (1906–2005) [19, 20]. Thus, this study focused on climate anomalies (variability) in different months as keys to affect malaria transmission dynamics in highlands as tried to be explained before in the study area [9].

The reason why 2000 m altitude became the upper limit of malaria transmission in the past might be related to the splenomegaly study conducted on children in different areas of Ethiopia in the 1950s [21]. The second reason was due malaria seasonality and transmission limited to certain months of the year [10, 22]. But, the study conducted at the household level on human settlements in highland areas of Ethiopia showed the existence of malaria transmission in all altitude ranges with a gradual decline in transmission (3.2%) in people living at 2500–3000 m altitude range [23]. In Debark district, 2800 m altitude was predicted to be the upper limit for the existence of malaria transmission [24]. In the absence of efficient vectors like *An. arabiensis* in very high altitude above 2000 m altitude, Myzomia species like *Anopheles demeilloni* and other paramyzomia species could play role in malaria transmission [24, 25]. Further investigation on potential vectors in highlands, in future, will answer many research gaps.

In the study area, malaria transmission was found to be perennial (continuous) with two peak transmissions (April—June, and October/November) following the two rainy seasons and lowest transmission during January–February dry season as described previously [8, 9]. This made the frequency distribution of mean monthly malaria counts over-dispersed for the different months of the years. The Poisson and Negative binomial regression models were recommended for count data like number of malaria cases in different time and space [27]. Although Poisson regression model is very suitable for modeling count data, one of the problems of Poisson regression is that it is affected by overspreading. The negative binomial regression model is normally used to accompany the Poisson regression especially when it suffers from the problem of over-dispersion. The aim of this study was to describe malaria transmission dynamics in GZD by comparing malaria case counts and incidence obtained from HPCs located at different altitude ranges, months and years using different statistical analysis including Poisson regression and negative binomial regressions. So far, no such kind of study has been conducted in Ethiopia or northwest Ethiopia which evaluated malaria transmission dynamics at different health facilities using appropriate statistical methods. By doing these objectives, the result has evaluated the malaria situation for 2020 and 2021, the years after phase one malaria elimination strategic plan has been completed. The results of this study will give a good insight about highland malaria transmission dynamics for the ongoing malaria elimination program (phase two).

## Methodology

### Study Areas

**Fig. 1:**
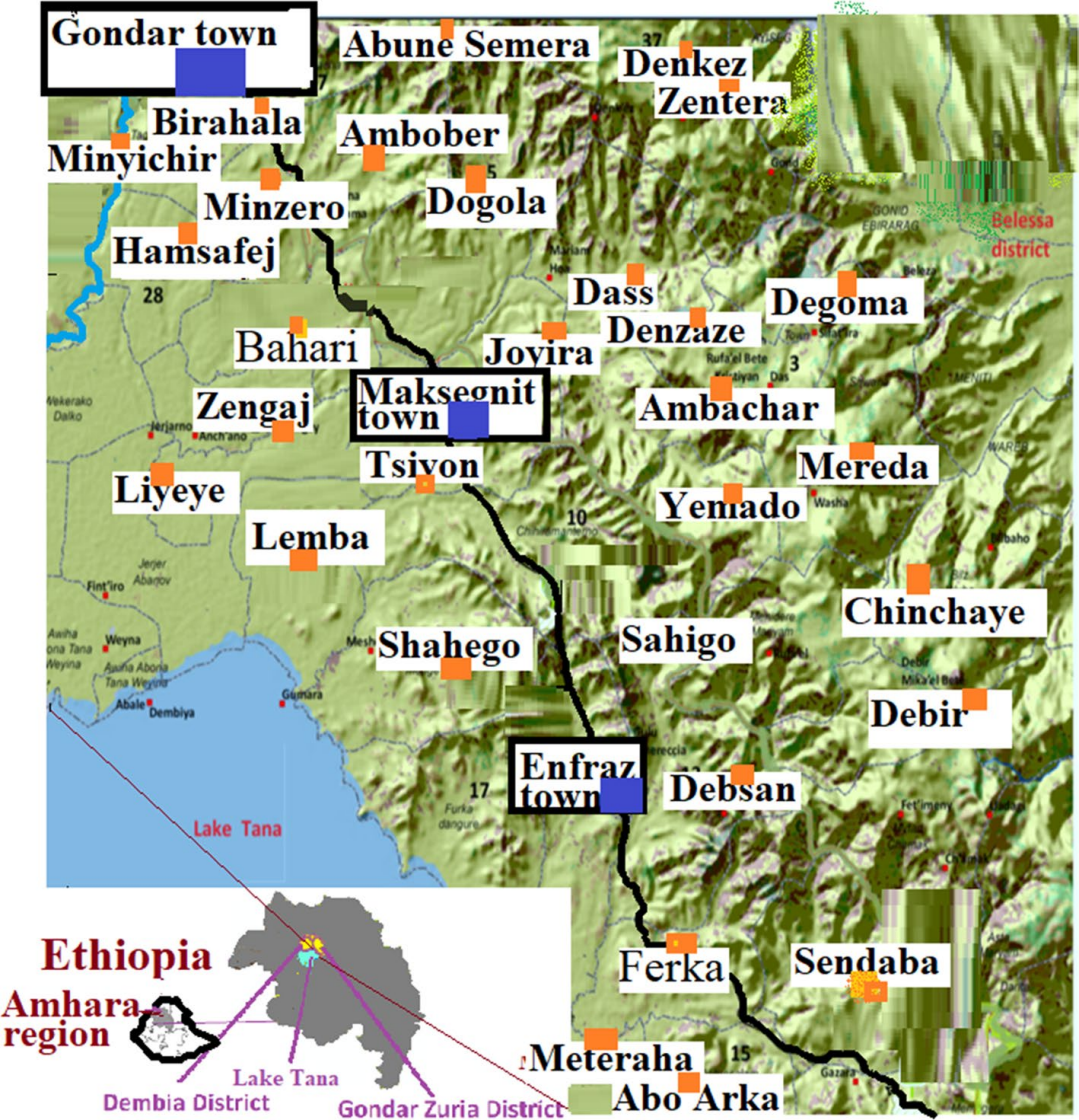
Map of Gondar Zuria district. The Map of Lake Tana and surrounding areas, which was used previously for biodiversity mapping, has been used after slight modification. ( Source: geoSYS Berlin, Germany www.geoSYSnet.de). Key: blue colored rectangle = towns; red colored rectangle = kebeles of rural villages

The Gondar Zuria District (GZD) study area is located on the northeast of Lake Tana in the direction of the road leading to Gondar town from Bahir Dar. The road leading to Gondar town from Bahir Dar town bisects the district. It is bordered by the Gondar town, Libokemkem district, and Belessa district in northern, southern, and eastern directions, respectively. The district is also bordered Dembia district in the western direction. The capital city of the Gondar Zuria District is Maksegnit town with the Enfraze town located on the road leading towards Bahir Dar at around 25 km. Degoma, Ambober, Lemba, Meterha and Abune semera are bigger villages. The group of villages on the same localities is grouped to form Kebele (the smallest administrative unit in Ethiopia). The altitudinal range of the Gondar Zuria district ranges from around 1800 m above sea level (asl) for villages next to Lake Tana to around 2 770 m (asl) around Denkez and Zentera villages on mountainous kebele (Fig. 1).

### Study Design and Method of Data Collection

Retrospective data of confirmed and treated malaria cases for all kebeles were obtained from the Maksegnit district Health Bureau, malaria expert desk. The Public Health Emergency Malaria (PHEM) reporting papers and excels reports for confirmed and treated malaria cases were analyzed starting from July 1, 2018 to December 17, 2021 (3.5 years) for all the total of 43 kebeles rural health clinics and posts in GZD. Malaria cases are diagnosed using microscopy in the health clinics while Pan-Pv-Pf RDT (combo) is used in health posts. In the health Bureau documents, parasites were identified by species and patients identified in different age groups (under 5 years, 5–14 years, and above 15 years) for every malaria WHO week. The data were tallied to obtain meaningful information such as monthly and annual malaria count (incidence). Google search of weather conditions data from the global meteorological web site for Maksegnit district (https://www.worldweatheronline.com maksegnit-weathe) has provided monthly mean for Average (Av.T), minimum (Min. T) and maximum (Max. T) temperatures in addition to Humidity and rainfalls for the different years. Careful analysis of climatic variables in relation to malaria counts at kebele and HPC clusters level were conducted with special emphasis on the effect of zero-month lagged rainfalls (RF), 1-month lagged rainfalls (RF1) and 2 months lagged rainfalls (RF2) on monthly malaria counts and incidences similar to the previous studies (6).

### Statistical Analysis

Poisson regression and Negative binomial log link models has been used to show a correlation between malaria count and monthly malaria transmission in addition to altitude ranges, years, and HPC clusters. Kruskal–Wallis tests are also used to see the existence of significant differences in malaria counts (incidences) in different kebeles and HPC clusters including altitudinal ranges. *p* values below 0.05 were considered as statistically significantly different districts. IBM SPSS Statistics software (version 25) was used for the analysis of the data.

### Quality Control

In an effort to reduce malaria-related death to near zero, health personnels in the districts of Maksegnit health bureau facilitates the malaria screening and treatment process seriously. Malaria screening and treatment are conducted for free in all government health facilities.

## Results

A total of 71, 928 confirmed malaria cases (54, 519 *Plasmodium falciparium* (75.8%) and 17, 409 *Plasmodium vivax* (24.2%) were treated in Maksegnit (13,574), Enfraz (21, 027), Meterha (12, 441), Lemba (13, 981), Minzero (8, 408), Ambober (507), Degoma (1, 632) and Abune Semera (358) HPC clusters from July 1, 2018 to December 17, 2021. The prevalence of malaria cases in GZD district during 2019, 2020 and 2021 were 2.4%, 13,2% and 13.9%, respectively. Of the total 54, 519 total *Plasmodium falciparium,* 37, 652 (69.1%) was identified using microscopy in the health clinics compared to 16, 867 (30.9%) diagnosed using RDTs. Similarly, of total 17, 409 total *Plasmodium vivax,* 12, 460 (71.6%) was identified in health clinics using microscopy compared to 4,949 (28.4%) identified using RDTs. Of overall malaria cases, 11.2% were reported from under 5-year-old children with the highest in Enfraz health posts and clinic cluster (13.96%) compared to the lowest (0.5%) in Degoma and Abune Semera. Malaria count (annual incidence) was 5, 893 (24 cases/1000 people) in 2019 compared to 31, 550 (131.97 cases/1000 people) in 2020 and 33, 248 (139.08/1000 people) in 2021. Mean monthly malaria incidence/1000 people in 2019 was 2.39 ± 5.4 and increased to 10.64 ± 16.99 in 2020 and 11.19 ± 16.59 in 2021.

When all kebeles assumed to have similar monthly rainfall, humidity and temperature parameters, spearman’s correlation indicated positive correlations between mean monthly incidences and one-month lagged rainfall (RF1) (*r* = 0.062^**^; *p* = 0.000), two-month lagged rainfalls (RF2) (*r* = 0.143^**^; *p* = 0.000), mean RFs (RF + RF1 + RF2/3) (*r* = 0.114^**^; *p* = 0.000) and humidity (*r* = 0.95^**^; *p* = 0.000) compared to negative correlations between mean monthly incidences and Maximum Temperature (*r* = − 0.054^*^; *p* = 0.000), Average Temperature (*r* = − 0.210^**^; *p* = 0.000) and Minimum temperature (*r* =− 0.269^**^; *p* = 0.000).

### Malaria Transmission Dynamics in Clusters of Health Posts and Clinics

**Fig. 2:**
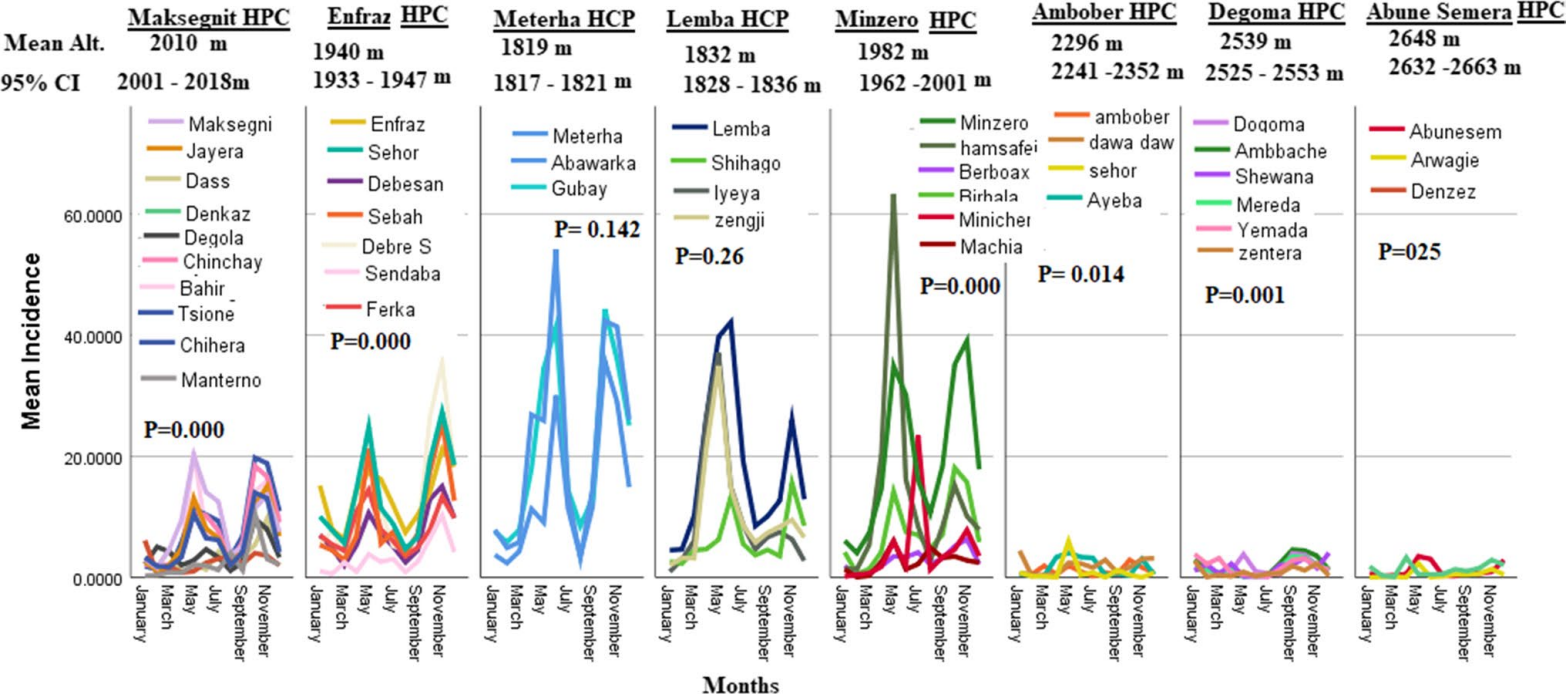
Malaria means monthly incidence/1000 people in different Health posts and clinics clusters during 2018–2021

During 2018–2021 study period, when the overall monthly mean ± Sd malaria incidence/1000 people for health posts and clinic clusters were compared, the highest (19.52 ± 26.5) was found for Meterha HPC and followed by Lemba (mean ± Sd = 10. 97 ± 17.42) (Fig. 2). Except Ambober–Abunesemera (*p* = 0.325), Maksegnit–Minzero (*p* = 0.225), Minzero–Enfraz (*p* = 0.374) and Meterha–Lemba (0.773) HPCs, all other pairwise HPCs comparison of malaria cases showed statistically significant differences (*p* < 0.005) during the study period.

Over all monthly malaria incidence per 1000 people during the study period for 1800–2000 m, 2001–2200 m, 2201–2400 m, 2401–2600 m and 2601–2800 m altitude ranges were statistically significantly different (*p* = 0.000). Of all pairwise comparison of the altitudinal ranges, only altitude ranges 2001–2200 m/2201–2400 m (*p* value = 0.224) and 2201–2400 m/2401–2600 m (*p* value = 0.218) showed no statistically significant difference for mean monthly malaria incidences per 1000 people during the study period. The wider altitudinal ranges which divided the study area into three altitudinal ranges (≤ 2000 m, 2000–2500 m and ≥ 2500 m) also showed statistically significant differences (*p* = 0.000) for mean monthly incidence/1000 people. When mean monthly malaria incidence among kebeles contained in the health posts and clinics clusters were compared, only the kebeles in Meterha (*p* = 0.142) and Lemba (*p* = 0.26) showed no statistically significant differences (Fig. 2). Going from 1800 to 2000 m altitude range to 2001–2200 m resulted in mean monthly malaria incidences sudden reduction from 10.62 to 3.8 similar to mean monthly malaria count which reduced from 64.66 to 15.5 during 2018–2021 study period.

All the kebeles in the Abune Semera HPC cluster are located on the mountainous areas (2648 ± 89.76 m altitude with the highest Denkez kebele (2772 m). In the Abune Semera HPC mountainous kebeles, a total of 358 confirmed malaria cases (Abune Semera health clinic (166), Denkez (114), and Anwagie (78) posts were treated from July 1, 2018–December 17, 2022. The highest annual malaria incidence (1.4 ± 2.03) was found in 2021. Generally, monthly malaria incidence in Abune Semera was lower compared to the other HPCs. The highest mean monthly incidence (5.06 ± 4.3) for Abune Semera recorded in November, 2020 and was higher compared to the corresponding incidences some of the HPCs located at lower altitude range (Fig. 2).

Similarly, Kebeles in Degoma HPC are located in the mountainous areas (2539.3 ± 112.56 m asl) with the Zentera (2770 m) kebele on the top. A total of 1632 confirmed malaria cases were treated in the all health facilities (Degoma kebele health clinic (273) and Amba Char (501), Mereda (337), Shewana (190), Yemeda (248) and Zentera (83) kebeles health posts) during the study period. The mean monthly malaria incidence ± Sd was 1.58 ± 1.90. Machia kebele health post (2290 m) is the only kebele found in 2201–2400 m altitude range. It is clustered in Minzero HPC cluster. In Ambober HPC (2296.7 ± 314.1 m altitude, a total of 507 confirmed malaria cases were treated (Ambober (181), Dawa (204), Sehor (27), and Ayeba (81). Ayeba has located on the highest (2729 m) mountains in Ambober HPC clustered kebeles. A total of 8408 confirmed malaria cases were treated in Minzero HPCs which include Berboax (5, 59), Birhala (Bichemer) (1, 594), Hamsafeji (2, 010), Macha (185), Minyichir (318), and Minzero (3, 742) were the kebeles where cases were treated during the study period. A total of 13, 574 cases treated in Maksegnit HPCs including Maksegnit ( 5, 505), Enfraz, Joyera (870), Dass (756), Denzez (264), Degola (478), Chinchaye (1, 345), Bahir (1, 721), Tsione (1, 194), Chihera(1, 197) and Menterno (244). The total malaria cases treated in Enfraz HPC cluster was 21, 027 including Enfraz clinic (5, 872), Sehor (4, 140), Debesan (2, 097), Sebah (3, 071), Debre selam (2, 064), Sendaba (924), and Ferka (2, 743). The remaining cases were treated in the Lemba and Meterha clusters.

Compared to mean monthly count for year 2021, Poisson and Negative binomial regressions models indicated 0.173 and 0.190 times lower malaria cases in 2019, respectively. In another words, 5.78-fold malaria cases increase for Poisson regression model or 5.26-fold malaria cases increase for negative binomial regression model in 2021 indicated compared to 2019. When the results of the two models were compared Poisson regression model estimation was very close to the real mean monthly count for 2021(Table 1).

**Table 1:** Comparison of Poisson and Binomial regression models to compare variation in mean monthly counts due to different years, altitude ranges, health facilities and months during 2018–2021

Poisson regression					Negative binomial reg		
Parameters	Mean monthly count	B(Log)	Sig.	Exp(B)	B(Log)	Sig.	Exp(B)
(Intercept)		1.627	0.000	5.087	1.944	0.000	6.986
1. Years: 2018	5.1	− 2.737	0.000	0.065	− 2.367	0.000	0.094
2019	11.42	− 1.754	0.000	0.173	− 1.661	0.000	0.190
2020	61.86	− 0.074	0.000	0.929	− 0.232	0.000	0.793
2021	65.97	0^a^		1	0^a^		1
2. 200 m altitude intervals: 1800–2000 m	64.66	3.200	0.000	24.528	2.784	0.000	16.191
2001–2200 m	15.5	1.783	0.000	5.951	1.414	0.000	4.111
2201–2400 m	4.4	0.504	0.000	1.655	0.526	0.013	1.692
2401–2600m	6.5	0.888	0.000	2.430	1.254	0.000	3.505
2601–2800m	2.7	0^a^		1	0^a^		1
3. Eth. FMOH intervals: 1750–2000 m	65.08	2.655	0.000	14.223	2.129	0.000	8.407
2001–2500 m	11.5	0.951	0.000	2.587	0.650	0.000	1.916
> 2500 m	4.51						
4. GZD HPC Clusters: Maksegnit HPC	32.36	2.46	0.000	11.72	2.27	0.000	9.72
Enfraz HPC	71.46	3.22	0.000	25.012	2.97	0.000	19.507
Meterha HPC	99.16	3.547	0.000	34.706	3.15	0.000	23.334
Lemba HPC	83.27	3.37	0.000	29.15	3.287	0.000	26.75
Minzero HPC	33.51	2.46	0.000	11.73	2.34	0.000	10.397
Ambober HPC	3.300	0.23	0.001	1.268	1.436	0.000	4.206
Degoma HPC	6.48	0.819	0.000	2.268	1.436	0.000	4.206
Abune Semera HPC	2.857	0^a^		1	0^a^		1
5. Months for 2018–2021:January	17.56	− 1.131	0.000	0.323	− 1.193	0.000	0.303
February	12.31	− 1.485	0.000	0.226	− 1.512	0.000	0.303
March	15.55	− 1.252	0.000	0.286	− 1.274	0.000	0.280
April	40.1	− 0.348	0.000	0.706	− 0.534	0.000	0.586
May	78.58	0.421	0.000	1.523	0.236	0.063	1.267
June	57.33	0.053	0.001	1.055	− 0.060	0.624	0.941
July	33.35	− 0.236	0.000	0.790	− 0.473	0.000	0.623
August	17.58	− 0.875	0.000	0.417	− 0.489	0.000	0.613
September	29.33	− 0.365	0.000	0.694	0.303	0.011	1.354
October	65.1	0.433	0.000	1.542	0.597	0.000	1.817
November	71.67	0.530	0.000	1.699	0.525	0.000	1.690
December	42.21	0^a^		1	0^a^		1
Scale		1^b^		1^b^			
Dependent variable: monthly count				1^b^			
a. Set to zero because this parameter is redundant							
b. Fixed at the displayed values							

Compared to 2601–2800 m altitude range, mean monthly count during the study period increased by 2.430 for 2401–2600 m altitude range, by 1.655 for 2201–2400 m, by 5.951 for 2001–2200 m and by 24.528 for 1800–2000 m using Poisson regression model. Similarly, negative binomial regression has indicated mean monthly count increase at different altitude ranges during the study period (Table 1). Poisson regression model estimated mean monthly counts at different altitude ranges, also, very close to the real values. The altitude related variation in mean monthly malaria cases in different health posts clinics clusters were also indicated in Table 1.

Compared to December using Poisson regression, malaria mean count increased by 1.7, 1.5, 1.1 and 1.5 in November, October, June and May, respectively. But, malaria count showed reduction by 0.7, 0.4, 0.8, 0.7, 0.3, 0.2 and 0.3 in September, August, July, April, March, February and January.

### The Role of Climate Variables on Mean Monthly Malaria Count (Incidence)

**Fig. 3:**
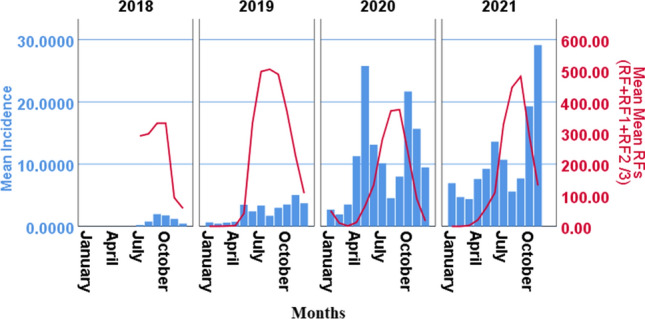
The relationship between mean monthly malaria incidence/1000 people plus mean monthly rainfalls to the different months during 2018–2021 in Gondar zuria district

Gondar zuria district rainfalls were normally distributed (ball-shaped) from January to December with peak in August (Fig. 3). Thus, Gondar zuria district malaria transmission season can be divided into March–June Bulg or spring season, July–September heavy rain season, October–December low rainfall season and January–February dry season. In March–June Bulg or spring season, malaria counts (incidences) increased with the progress of rainfalls. Generally, increase in rainfall during this season had positive effect on malaria transmission. The linear increase usually interrupted by the heavy rain at the end of June. During July–September heavy rain season, the extent malaria transmission was affected by the intensity of rainfalls which could kill the mosquito immature stages. During this heavy rainfall months, relative increase in rainfalls had negative effect on malaria transmission. October–December low rainfall season favored high transmission when the rainfalls in this season were relatively low. Malaria transmission in January–February dry season was dependent on the presence of rivers in the highland areas. In northwest highland Ethiopia, proximity to the river was the base for human settlements. Thus, low transmission in dry season was maintained by the presence of rivers which served as habitat for malaria vectors’ immature stages. During the study period, January–February dry months resulted in the least (3824) malaria counts while the highest was found in October–December low rainfall season (30, 330). The second high malaria transmission was found in March–June Bulg/spring season (24, 129). A total of 13, 645 malaria cases were counted in July–September heavy rain months (Fig. 3).

In 2019, the relatively high annual mean rainfall (Mean ± Sd = 195.93 ± 208.8) produced 5,893 malaria cases compared to the least rainfall (130.98 ± 163.89) in 2020 which produced 31,550 malaria cases. The relatively moderate rainfalls in 2021 (161.42 ± 211.54) produced the highest malaria cases (33,248). The distribution of monthly rainfalls in 2019, however, was not in a way to favor malaria transmission. Very low rain in Bulg/spring season and heavy rains in summer season which extended until November, most probably, affected the survival of the immature stages. The monthly distribution of the mean rainfalls had operated, most probably, in a way that disfavored malaria transmission in 2019 compared to 2020 or 2021. Very low rainfalls in March (2.1 mm), April (4.00 mm) and May (117.4 mm) in 2019, compared to highest March (8.2 mm), April (48.03 mm) and May (147.7 mm) rainfalls in 20,120, most probably, did not favored malaria transmission in Bulg/Spring in 2019. Thus, very low total malaria case counts (1340) were found during March–June Spring/Bulg season in 2019 compared to higher counts in 2020 (14,006) and 2021 (8,783). Sudden decline in malaria transmission is usually started from heavy rain in June due to killing effects of the heavy rainfalls and flooding during heavy rain months. Rainfalls in June, 2019 (438.7 mm) was greater compared to June, 2020 rainfall (151.8 mm) or June, 2021 rainfall (265.3 mm). This might have also contributed for much lower July–September, 2019 counts (1398) compared to July–September 2020 (5, 410) and 2021 (6,163) malaria case counts (Fig. 3).

The difference in malaria incidences in relation to rainfall variability in the different years could be explained based on their difference on zero- to two-month lagged rainfalls. This graph was drawn by considering the effect of 0, 1 and 2 months lagged rainfalls (RF + RF1 + RF2 divided by 3).

## Discussion

It is indisputable about the role of climate variability (rainfall and temperature) on the transmission dynamics of malaria in highland areas [28, 29]. The impact of climate factors on malaria transmission could be seen year after year with pronounced effect during EL- Nino/La-Nina years in Ethiopia, especially, northwest highland areas where malaria transmission dynamics seemed strongly affected by rainfall variability [9, 12]. For example, the number of confirmed Ethiopian malaria cases in 2010 (1480, 306 cases) was less than the number of cases (1, 530 739 cases) in 2017 [30]. This does not mean that the interventions conducted for 8 years (2010–2017) did not avert malaria incidences or reduced malaria-related deaths. In a country like Ethiopia where climate variability (anomalies) produced unpredicted malaria incidence, interventions play significant roles in reducing the effect of malaria-related mortality and morbidity, especially, in high malaria transmission years. Until the report of 1, 743, 755 malaria cases for 2020 [4], Ethiopia was reported as country which showed 40% reduction from what was in 2015 base line cases (1, 867 058) [1, 3]. In the study area, malaria cases in 2020 also increased to 31, 550 from what was in 2019 (5893). The increase, however, was very high (5.35 fold increase). Most probably, sudden increase in malaria cases in highland malaria-endemic areas, including the study area, have played significant role in the recent (2020) increase in Ethiopia [4]. For 2021, in the study area, the reported malaria count (33, 248) was greater than what was in 2020 (31,550). Most probably, interruption of the use of malaria control tools in the 2020 and 2021 and malaria transmission favoring mean monthly rainfall patterns [9] in these years had played significant role for the sudden increases. If Ethiopian malaria case remained high or greater than from what was in 2020 and 2021, Ethiopia will be out of the track of achieving malaria elimination goal. Malaria transmission in the study area (Ethiopia) during 2020 and 2021 alarming danger for prompt interventional measures.

Altitude and temperature are closely linked variables. If there is an increase in altitude, obviously, there will be a corresponding decline in temperature. It was estimated that for every 100 m increase in altitude, there will be a corresponding 0.5 °C decline in temperature [28]. When altitude decrease, temperature increase which accelerates the sporogonic cycle of malaria parasites in the vectors (malaria transmission) and vis-versa [29, 31]. It is not surprising if sporogony is delayed in malaria vectors with lower temperature during laboratory experiments [32] as it works for all enzymes which require an optimum 37 degrees centigrade for optimum action. For example, of all malaria cases during the study period, 36.7% (26,422) was counted from relatively hotter Meterha (95% CI = 1817–1821 m) and Lemba HPCs (95% CI = 1828–1836 m) located near Lake Tana (Fig. 2), In these relatively hotter villages, *Anopheles arabiensis*, *Anopheles pharonensis* and *Anopheles funestus*, most probably, is playing a major role in malaria transmission (33). In very high altitude (relatively low temperature), beyond the distribution range of *Anopheles arabiensis*, less efficient vectors (*Anopheles cinereus* and different Myzomia species) could serve to transmit malaria at a lower rate [25]. As it can be seen from our data, highland malaria transmission was consistent in very high altitudes including Kebeles in 2601–2800 m (Fig. 2).

Altitude (temperature) connected decline in mean malaria incidence was clearly observed with the implications of temperature covariates (Maximum, minimum and average temperatures) playing major role in highland malaria transmission dynamics in space and time. But, the luck of meteorological stations in all the 43 kebeles in the districts could not allow us to compared the direct effect of temperature covariates. When spearman’s correlation test was conducted, statistically significant positive correlations between mean monthly malaria incidence and rainfall parameters (*r* ≥ 0.063**; *p* ≤ 0.009) and negative statistically significant correlation between mean monthly malaria incidence and temperature parameters(*r* ≤ − 0.054; *p* =− 0.024–0.000) were found. But, careful interpretation of these spearman’s correlation results are required as there is a compounding effect of temperature during dry season as described previously [9]. In the study highland area where the temperature ranged from 10 to 30 °C_,_ there may not be temperature-related death of the immature stages in the aquatic habitats and malaria parasites death in the gut of the parous female mosquito vectors in human dwellings or in other shelters. The impact of temperature is, therefore, related to slowing dawn transmission during low temperature. This has been seen clearly by using Poisson and Negative binomial regression models which compared the increase in malaria transmission when altitude decreases (Table 1). On the other hand, high intensity of rainfalls and the associated flooding affects the survival of the immature stages and indirectly affecting the rates of malaria transmission in different months. Poisson and negative binomial regression models also analyzed the rate of malaria transmission in different months and years.

Using altitude range measurements, it was possible to estimate the rate of malaria transmission in different altitude ranges. Compared to 2601–2800 m altitude range, malaria transmission in 1800–2000 m altitude range was higher by 24.5 and 16.2 times for Poisson and Negative binomial regression models, respectively. When the two models was used together for the five parameters we analyzed (Table 1), Poisson model B(exp) values have helped to estimate malaria mean counts almost equal to the true values. Thus, Poisson regression analysis is better statistics to estimate rate of malaria transmission in time and space (Table 1). Although malaria transmission declined suddenly from 64.66 mean counts at 1800–2000 m to 15.5 at 2001–2200 m, altitude 2000 mwas not found as cutoff for malaria transmission as previously reviewed by Cox et al. [22].

Naturally, there is no control tool that is 100% effective and stops malaria transmission. In the absence of a 100% effective control tool to stop malaria transmission, one of the challenges in the malaria elimination program in Ethiopia is, again, the difficulty to separate whether malaria incidence increase or decrease was due to the interventions or due to the impact of high or low anomalous climatic variables. In Ethiopia, where highland Kebeles above 2000 m were not part of the malaria elimination program, it is not possible, perfectly, to compare malaria count (incidence) of altitude below 2000 m and above 2000 m. Although it might be difficult to tell exactly the source of recent increase in malaria counts in the study area or Ethiopia, increase in malaria transmission is an implication for a failure in malaria elimination program and should be considered seriously. Most probably, interrupting malaria control tools in 2020 and 2021 and malaria transmission favoring mean monthly rainfalls in different months worked synergistically to bring about the current sudden increase in malaria incidences in the study areas.

## Conclusion

Based on the current situation, the Ethiopia malaria elimination program cannot conduct surveillance to track, diagnose and treat malaria cases for affordability and cost-effective reasons. The number of malaria cases, particularly, in northwest Ethiopia is very high. It is necessary to focus on tracking to understand the malaria epidemiology. There should be surveillance for anomalous climate variables in relation to malaria transmission for immediate malaria interventional actions. Interruption of the use malaria control tools before reaching the pre-elimination stage in Ethiopia may return malaria situation back to high transmission in most of the country. Evidence-based cost-effective environmentally friendly sustainable use malaria control tools are recommended until malaria elimination stage is reached.

## Data Availability

The data are available from the corresponding author and can be shared at reasonable request.

## Abbreviations

ITNs: Insecticide-treated nets
WHO: World Health Organization
GZD: Gondar Zuria district
HPC: Health posts and clinic
PHEM: Public health emergency malaria
Av.T: Average temperature
Min.T: Minimum temperature
Max.T: Maximum temperatures
RF: Zero-month lagged rainfalls
RF1: 1-Month lagged rainfalls
RF2: 2-Month lagged rainfalls
